# Supplementary material for: Multitarget Potential of Phytochemicals from Traditional Medicinal Tree, Terminalia arjuna (Roxb. ex DC.) Wight & Arnot as Potential Medicaments for Cardiovascular Disease: An In-Silico Approach
Source: Molecules. 2023 Jan 20;28(3):1046. doi: 10.3390/molecules28031046 (PMC9920080; doi:10.3390/molecules28031046)
Supplement: Supplementary file 1 [file molecules-28-01046-s001.zip › molecules-2126927-SI.pdf]

**Table S1:** Binding energy of re-docked ligands from *T. arjuna* against targeted protein receptors.

| PDBs | Native ligands | Binding energy (kcal/mol) |
|---|---|---|
| 4YAY | ZD7 | -4.565 |
| 4DLI | IRG | -5.654 |
| 1HW9 | SIM | -18.276 |

**(A)** **(B)**

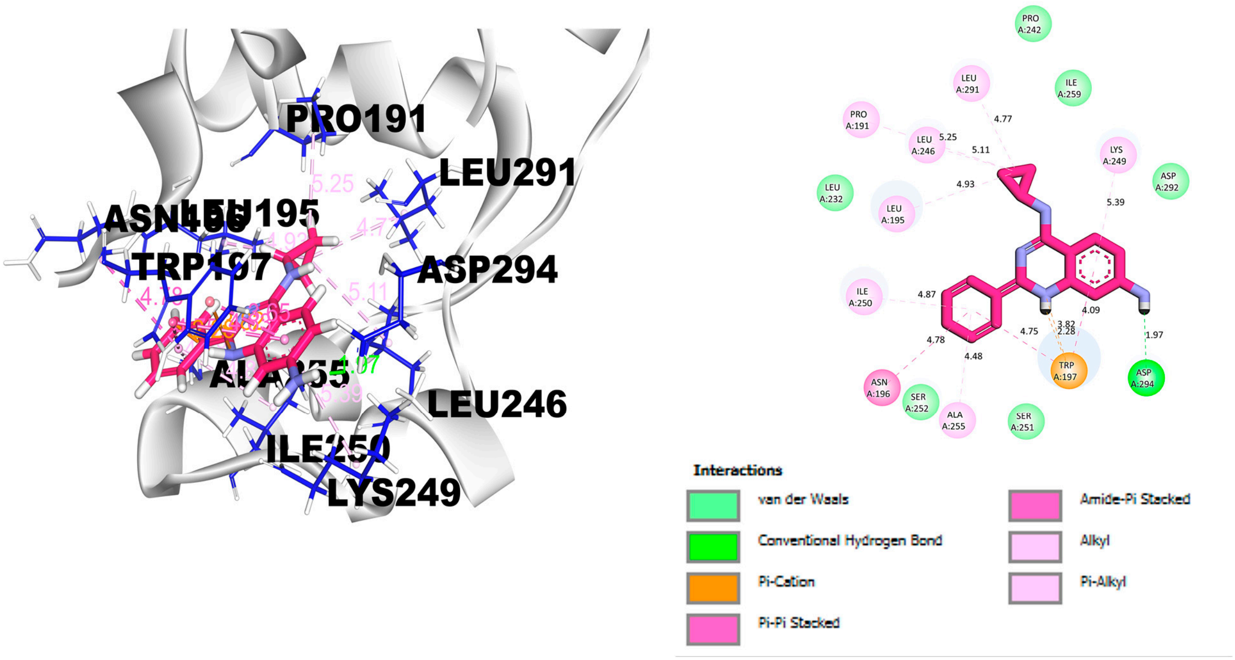

**(C)**

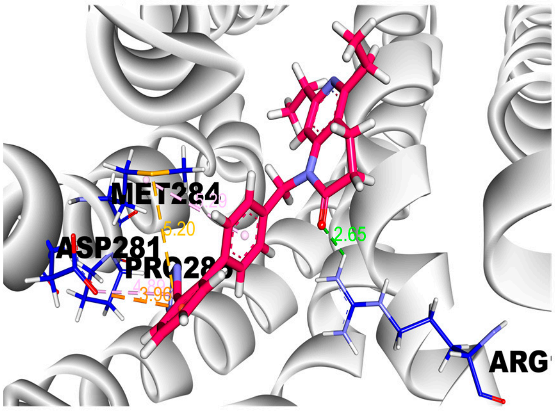


**(D)**

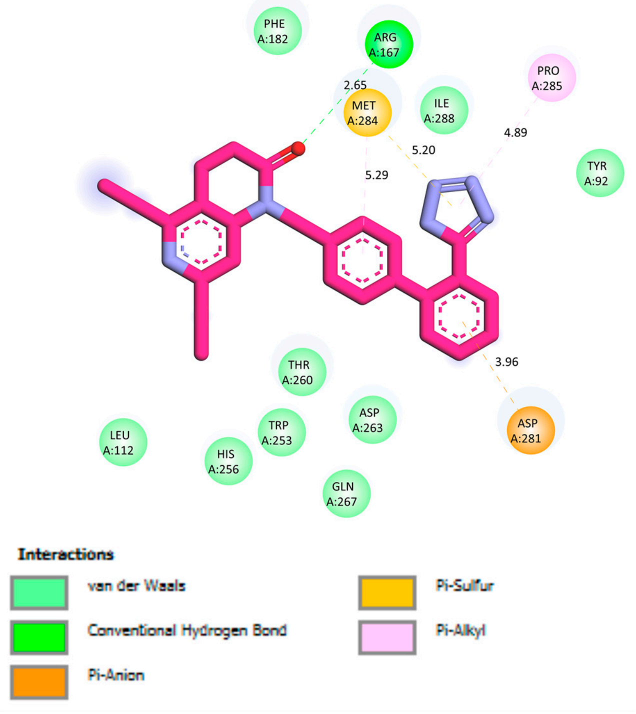


**(E)**

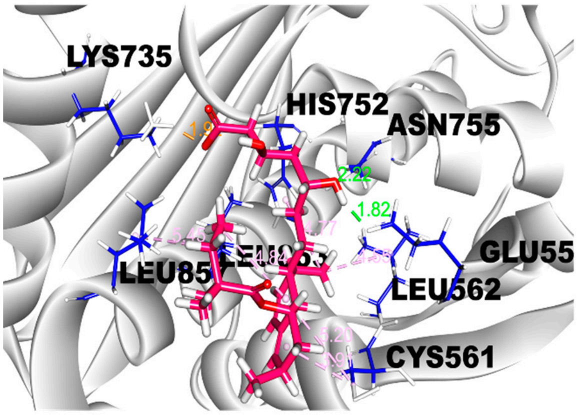


**(F)**

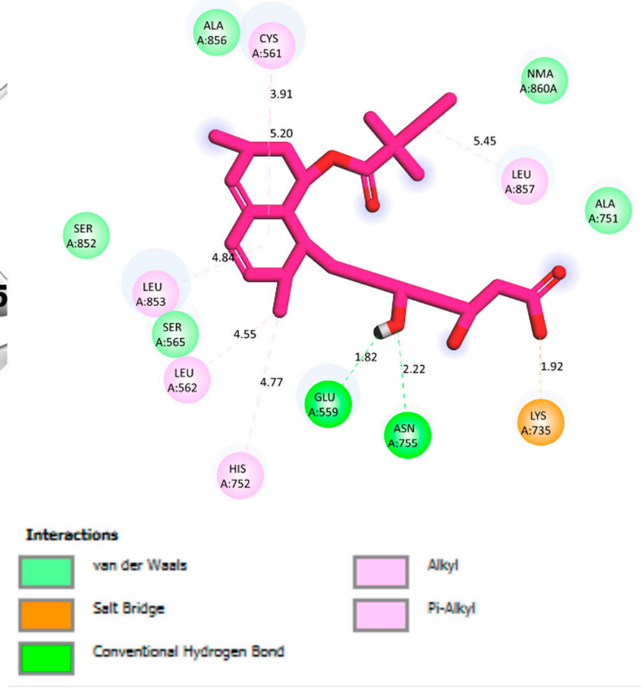


**Figure S1:** Docked pose of the native ligand with target proteins. (A) 3-D interactions of native ligand with interacting amino acids of 4DLI; (B) 2-D interactions of casuarinin with interacting amino acids of 4DLI; (C) 3-D interactions of native ligand with interacting amino acids of 4YAY; (D) 2-D interactions of native ligand with interacting amino acids of 4YAY; (E) 3-D interactions of native ligand with interacting amino acids of 1HW9; (B) 2-D interactions of native ligand with interacting amino acids of 1HW9.
